# Unified modelling of gaze and pupil dynamics in saccadic tasks

**DOI:** 10.1038/s41598-026-51489-9

**Published:** 2026-05-01

**Authors:** Julián Espinosa, Marta Guisot, Aurora Larrosa, Jorge Pérez

**Affiliations:** 1 Department of Optics, Pharmacology and Anatomy, Universidad de Alicante, Carretera San Vicente del Raspeig, s/n, San Vicente del Raspeig, 03690 Alicante, Spain; 2 Instituto Centro de Investigación Operativa, Universitas Miguel Hernández, Avenida de la Universidad, s/n, Elche, 03202 Alicante, Spain; 3 University Institute of Physics Applied to Sciences and Technologies, Universidad de Alicante, Carretera San Vicente del Raspeig, s/n, San Vicente del Raspeig, 03690 Alicante, Spain

**Keywords:** Saccadic eye movements, Post-saccadic oscillations, Pupil area variation, Gaze dynamics, Neuroscience, Psychology, Psychology

## Abstract

This study introduces a unified phenomenological framework to characterize the interconnected dynamics of gaze displacement and pupillary area variation during large-amplitude (30°) horizontal saccades. Using a high-resolution dataset from 242 healthy participants, we modeled primary saccadic shifts and post-saccadic oscillations (PSO) using a combined sigmoidal Boltzmann and damped harmonic oscillator approach. Our framework demonstrates that even after correcting for pupil foreshortening error, the pupillary signal contains physiological PSO-like oscillations that mirror gaze dynamics—a phenomenon previously uncharacterized in the literature. To account for the non-normal, heavy-tailed distribution of oculomotor parameters, population characteristics were defined using t location-scale modeling. Non-parametric Wilcoxon signed-rank tests revealed significant directional asymmetries; specifically, leftward movements exhibited longer total durations (∆*t*) and slower stabilization compared to rightward shifts. However, mechanical parameters such as oscillation amplitude and gaze baseline offsets remained directionally invariant (*p* > 0.01), suggesting a decoupling between asymmetric neural motor preparation and symmetrical biomechanical constraints. By establishing high-precision baseline parameters in an extensive population, this model provides a foundation for using joint gaze-pupil signatures as sensitive biomarkers for detecting subtle oculomotor and autonomic dysfunctions.

## Introduction

The ability to direct gaze rapidly and accurately is fundamental to visual perception, attentional control, and motor coordination. Saccadic eye movements, which allow for rapid shifts in gaze between points of interest, are closely linked to pupillary responses that dynamically adjust based on cognitive processing, attentional shifts, and motor preparation. Both mechanisms share overlapping neural pathways, highlighting the interconnected nature of gaze dynamics and pupil modulations^1–5^. Investigating these interactions provides valuable insights into how visual attention and motor control are integrated, with implications for basic neuroscience, clinical research, and technological applications.

In this study, the analysis is specifically focused on visually-guided, horizontal saccades of large amplitude (30°). A distinction is made between these and other oculomotor phenomena, such as exploratory saccades occurring during free-viewing or involuntary microsaccades that occur during fixation. By utilizing high-amplitude, task-driven saccades, the fine-scale dynamics of both the primary gaze shift and the subsequent PSO can be more effectively isolated and modeled. While large-amplitude saccades provide a robust signal for modeling, it is acknowledged that their dynamics may differ from smaller or more spontaneous saccades, which are often executed with different ‘braking’ mechanisms and cognitive demands.

Recent research suggests that pupillary dynamics serve as key markers of motor preparation, with pre-saccadic pupil dilation occurring before eye movement initiation^4,6–9^. Such pre-saccadic modulations are consistent with evidence from animal models, where pupil dilation has been observed to precede self-initiated motor behaviors like locomotion and whisking^10–12^. These observations suggest that pupillary changes may reflect a generalized arousal-related signal associated with motor preparation rather than being a purely sensory or reactive phenomenon. Consequently, il signal is increasingly viewed as a proxy for the internal state of the oculomotor system during the planning phase of a gaze shift. This anticipatory response indicates that pupil size is not solely reactive to luminance but is also influenced by higher-order cognitive functions such as decision-making, attention allocation, and motor readiness^13–15^. Despite these findings, the precise relationship between gaze displacement (GD) and pupillary area variation (PAV) requires further investigation to clarify their coordination and underlying neural mechanisms.

A fundamental aspect of saccadic eye movements is their hemispheric control, which influences differences between rightward and leftward saccades. Typically, the left hemisphere predominantly controls rightward movements, while the right hemisphere controls leftward movements, leading to functional asymmetries^16^. Rightward saccades tend to adapt more efficiently to visual cues, while leftward saccades exhibit greater stability but slower adaptation^17,18^. Beyond innate wiring, it is possible that habitual motor patterns, such as the repetitive leftward return sweeps required by Western reading habits, further refine these dynamics and their underlying hemispheric modulation. Such experience-dependent adaptation could prioritize stability in leftward movements to ensure precise positioning at the start of a new line. By modeling these asymmetries, the relative contributions of neural control and acquired habits to the stability of the gaze-pupil relationship can be better characterized.

Saccadic eye movements often exhibit PSO, characterized by a brief period of instability where the eye settles upon reaching the target. Rather than representing deliberate corrective saccades, this phenomenon is widely understood to reflect a biomechanical ‘settling’ process. It arises from the substantial initial force required to initiate saccades, resulting in damped oscillations as the eye stabilizes^19–21^. The magnitude and duration of these oscillations are directly linked to the forces acting on the eyeball, including orbital tissue resistance and rotational inertia^22–25^. In video-based eye-tracking, these oscillations may be further influenced by the internal dynamics of the eye, such as the inertial movement of the lens and pupil relative to the cornea. Beyond their role in stabilizing gaze, PSO serve as indicators of oculomotor function, particularly in neurological conditions^26,27^.

Video-based eye trackers are widely used to measure fixations, saccades, and PSO. A significant challenge in eye movement research is ensuring the accuracy of eye-tracking methodologies, particularly in saccade classification algorithms. Several widely used methods, such as the EyeLink Parser^28^, machine-learning-based approaches^29^, and the Minimal Norm Hessian (MNH) algorithm^30^, developed as an extension of Nyström and Holmqvist’s work^31^, aim to improve saccade detection. However, these techniques remain susceptible to misclassification errors, which can distort data accuracy.

Fixation misclassification is a common issue, where small fluctuations in the eye-tracking signal are mistaken for saccades, leading to an overestimation of saccade frequency. The variability of the speed and trajectory of the saccades causes segmentation errors whereby one saccade is erroneously divided into several smaller saccades^32–34^. Additionally, blinks and data loss frequently lead to false saccade detections, with algorithms incorrectly interpreting gaze reappearance after a blink as a new saccade^35,36^. Given the importance of accurate classification, this study aims to refine detection methodologies and improve the reliability of eye movement and eye-tracking research.

Another significant issue in eye-tracking studies is the pupil foreshortening error (PFE), which systematically distorts PAV measurements due to gaze angle changes^4,5,37,38^. As the eye rotates, the circular pupil appears elliptical, leading to an underestimation of pupil diameter. This effect becomes particularly pronounced in large saccadic movements (> 30 degrees visual angle), where PFE can cause up to 15% variation in measured pupillary area^37^. While our unified model addresses these distortions mathematically, alternative measurement strategies, such as selectively analyzing the pupil’s vertical diameter (the minor axis of the ellipse), could also mitigate these geometric artifacts. However, as the current study is based on an extensive existing database where raw ocular images were not preserved, such axis-specific analysis was not feasible. Our proposed modeling framework therefore provides a necessary analytical alternative for correcting foreshortening errors when raw geometric descriptors are unavailable. Incorporating such axis-specific metrics into future studies using raw image data could further enhance signal stability in high-eccentricity tasks. The present study delves into the analysis of PAV, aiming to determine whether this variation is solely due to PFE or if there is a genuine change in pupillary area that could be mistaken for this error, thereby improving the reliability of pupillometric research.

**Fig. 1:**
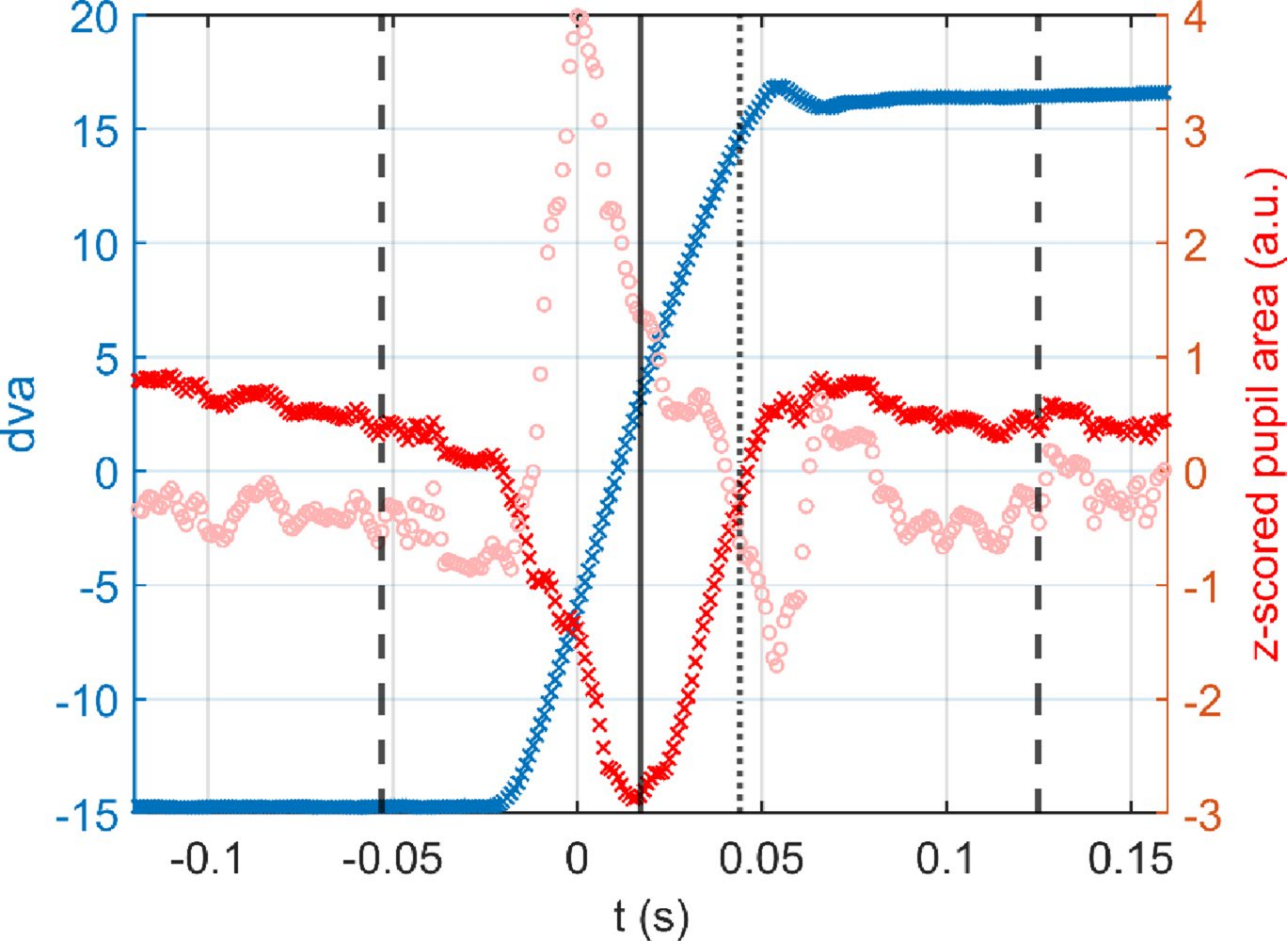
Procedural overview of signal segmentation and PFE-correction. This figure illustrates the raw input data and the temporal landmarks used for the analysis. Blue crosses denote GD, while light red circles represent z-scored PAV. The peak of z-scored PAV marks t = 0 s on the x-axis. Red crosses indicate z-scored PAV corrected of PFE, with its peak marked by a solid black vertical line. The vertical dashed, dotted, and solid lines represent the temporal boundaries (initial/final instants and peaks) used to segment the signal for subsequent modeling. To ensure methodological transparency, this figure displays the raw sampled data points to highlight the signal-to-noise ratio and the specific effects of the PFE-correction, rather than the resulting model fits described in Eqs. (2) and (3).

While human vision involves a variety of movement types, ranging from small exploratory saccades to corrective glissades, this study focuses on task-driven, large-scale saccades. These 30° movements are uniquely suited for investigating somatic-autonomic integration because their high inertial demands amplify the biomechanical and neural constraints of the oculomotor system. By focusing on these ‘stressed’ movements, we can more clearly observe the interplay between the motor command and the subsequent pupillary stabilization (as illustrated in Fig. 1), providing a high-resolution window into the neural effort that might be less visible during smaller, more routine exploratory shifts.

Beyond its immediate objectives, this study aims to establish a baseline for future research in both fundamental and clinical sciences. Many neurological disorders, including Parkinson’s disease, Huntington’s disease, and multiple sclerosis, exhibit distinctive saccadic and pupillary abnormalities^39–45^. For instance, Parkinson’s disease patients exhibit impaired saccadic damping, suggesting that PSO analysis could serve as a potential biomarker for oculomotor dysfunction^26^. Determining ranges of characterizing parameters of GD and pupillary responses during saccades and PSO in a healthy population is crucial for understanding the mechanism of gaze stabilization. This knowledge has important implications for neurological and clinical assessments and offers a foundational reference for diagnosing and monitoring oculomotor dysfunction.

Building on these objectives, this study proposes a unified phenomenological model of gaze and pupil dynamics in saccadic tasks to enhance the reliability of eye movement and pupillometric research through refined detection methodologies. The model is designed to characterize the observable temporal signatures of the gaze-pupil relationship without simulating the underlying muscular forces or specific neural firing patterns. It assesses whether the PFE may be masking genuine changes in pupillary area. Moreover, it establishes baseline parameters for GD and pupil dynamics during saccadic tasks in a healthy population, contributing to advancements in both fundamental and clinical sciences. Specifically, by investigating hemispheric differences in saccadic movements and analysing PAV, this study may help improve our understanding of the neural mechanisms underlying saccadic execution and pupillary responses.

## Methods

A recently created and freely distributed GazeBase (CC BY 4.0) database^46^ contains 12,334 monocular (left-eye) eye-movements recordings from 322 individuals, with normal or corrected-to-normal visual acuity, while performing seven discrete tasks. Eye movements were captured at a 1000 Hz sampling rate using an EyeLink 1000 eye tracker (SR Research, Ottawa, Ontario, Canada), a video oculography device that provides gaze locations, that are estimated from pupillary-corneal reflection vectors using a polynomial mapping developed during calibration, and uncalibrated PAV values in arbitrary units. Data obtained for the Horizontal Saccade Task (HSS) from 309 participants were assessed in this work. The HSS task was designed to elicit visually guided horizontal saccades of constant amplitude by periodically displacing a target on a 2680 × 1050 pixel monitor, positioned at 550 mm in front of participants’ chair. A bull’s-eye target, displayed on a black background, was displaced every second between two positions located ± 15° of the visual angle (dva) horizontally from the centre of the screen, thus ideally eliciting a 30-degree horizontal saccade with each jump displacement. The target remained in each position one second before transitioning, with a total of 100 transitions occurring during each recording.

The MNH algorithm was applied to detect and extract fragments of the sequences that comprise a saccade and PSO from the first 80 s of each sequence from the HSS database. To refine the extracted data, only saccades with amplitudes exceeding ± 13 dva were first selected, eliminating some potential misclassified instances from the algorithm’s output. GD and PAV were extracted within a window from 100 ms before the onset to 100 ms after the conclusion of the saccadic movement, as determined by to the MNH algorithm. Gaze dva horizontal and vertical components, *x* and *y*, respectively, were transformed to gaze dva displacement following (1).

1 $$\:\mathrm{GD}=sgn\left(x\right)\sqrt{{x}^{2}+{y}^{2}}$$

An example of a fragment extracted from a sequence is shown in Fig. 1. Blue crosses correspond to registered GD.

The pupillary data were processed by first removing the best-fitting straight line to eliminate trends. Subsequently, z-score normalization was applied by subtracting the mean and dividing by the standard deviation, standardizing the data to a mean of 0 and a standard deviation of 1. This rescaling allowed for comparisons across different sequences or participants. An example post-processed PAV around a saccade is also presented with light red circles in Fig. 1. Due to the projection of the pupil onto the camera sensor in different gaze directions, the recorded pupil area appears reduced when the gaze direction deviates from the camera axis. As a result, a peak in PAV is observed when this foreshortening effect is minimized. However, a secondary peak emerges after that initial response, that could not be attributed to PFE-induced artifacts and resembles post-saccadic oscillatory behaviour. This raises a key question: Are the observed PAV merely artifacts of PFE, or do they reflect genuine physiological responses?

PFE has been addressed in the literature using a geometric model that accounts for the foreshortening of the pupillary area, expressing the pupillary diameter variation as a function of the cosine of the angle between the eye-to-camera axis and the eye-to-stimulus axis^37^. Following that approach, registered PAV was PFE corrected just dividing it by the square cosine of the registered dva. The z-scored PFE-corrected data was presented in red crosses in Fig. 1. A pupil contraction and subsequent dilation is observed during the saccade, and an oscillation after those ones can also be identified.

While the current study utilizes a mathematical correction based on gaze eccentricity to account for PFE, it is acknowledged that alternative measurement strategies exist for mitigating geometric distortions. These include the selective analysis of the minor axis of the pupillary ellipse, which remains relatively invariant under horizontal rotation^47^, or the use of 3D eye models for dynamic area estimation^37^. However, for the 30° horizontal saccades analyzed here, the square-cosine correction was considered a sufficiently robust approach to isolate the underlying physiological modulation from the primary geometric artifact.

The proposed approach to model eye gaze and pupil size behaviour during the saccadic movement consists of using a sigmoidal Bolztman function shown in Eq. (2), which refers to the rapid shift of gaze direction from one point to another and typically follows a sigmoidal shape. Saccade is followed by PSO, whose characteristics can be modelled by a damped oscillation function plus a linear term^25^ presented in Eq. (3).

2 $$\:{f}_{1}\left(t\right)=\frac{a}{1+{e}^{-b\left(t-c\right)}}+d$$

3 $$\:{f}_{2}\left(t\right)=A{e}^{-\left(\frac{t-{t}_{0}}{\delta\:}\right)}\mathrm{cos}\left(\frac{2\pi\:(t-{t}_{0})}{T}\right)+g(t-{t}_{0})+h$$

The goal of this modelling approach was to determine whether PAV could be modelled similarly to GD, while also evaluating potential differences in their temporal characteristics. If both datasets can be effectively modelled using the same equations, it suggests that the phenomena may share similar descriptions, though this does not necessarily indicate a shared underlying mechanism. A common mechanism would only be implied if the parameters exhibited synchronous behaviour.

The stochastic nature of the parameters was evaluated by systematically testing twenty-three candidate probability density functions. We hypothesized that a TLS distribution would be more appropriate than a standard Gaussian model due to the observed presence of heavy tails and outliers in the empirical distributions. The final model selection was based on the Akaike Information Criterion (AIC) to ensure the most robust fit for the subject-level data.

The instant limiting saccade and PSO in the gaze trajectory can be roughly defined from the overshot peak which appears. A peak detection algorithm was employed to identify prominent peaks. Then, a window from 70 ms before the peak in the z-scored PFE-corrected PAV sequence (initial instant) to 110 ms after that peak (final instant) was set to fit the trajectory to Eq. (2), and another from the peak in PFE corrected PAV sequence to the final instant was selected to fit it to Eq. (3). These instants are respectively plotted with dashed and continuous vertical black lines in the example shown in Fig. 1. Regarding PAV sequences, an epoch from the initial instant to the instant of the peak in the z-scored PFE-corrected PAV sequence was selected for fitting Eq. (2), whereas another from this last to the final instant, or extending to the end of the sequence, was used for fitting Eq. (3). These epochs can be identified in the figure as the windows between the first dashed black line and the solid black line, and the window between the latter and the last dashed black line. Note that although these intervals may seem quite different from those selected for fitting GD data, the overall time window remained the same.

The model’s validity can be assessed based on the percentage of valid fits per subject, where a model fit is considered valid if the coefficients of determination of the fitting to Eqs. (2) and (3) are greater than a threshold value, 0.8 in this work. A high mean validity rate indicates the model is generally reliable. As long as the outliers remain a small minority, the model can still be considered valid despite some isolated deviations.

The distributional characteristics of the parameters derived from the fits to Eqs. (2) and (3) were initially assessed using Shapiro-Wilk tests to evaluate the assumption of normality. Given that eye-tracking data often exhibit high kurtosis and significant outliers, this step was critical for determining the most appropriate modeling and testing framework. Upon the identification of non-normal distributions, the parameter densities were modeled using a TLS distribution. This choice was made to provide a more robust characterization of the data, as the TLS distribution incorporates a shape parameter that accounts for the heavy tails typical of oculomotor signals. The selection of the TLS model over standard Gaussian or other heavy-tailed alternatives was validated through the comparison of AIC values.

Following the same logical framework, the comparison between leftward and rightward movements was conducted using non-parametric statistics. Wilcoxon signed-rank tests were employed to evaluate differences in parameter values based on saccade direction. These tests were performed at the subject level, utilizing the median value of each parameter per participant across all successful trials. This approach was adopted to ensure that the statistical inference remained robust against trial-to-trial variability and the stochastic nature of the PSO.

## Results

**Fig. 2:**
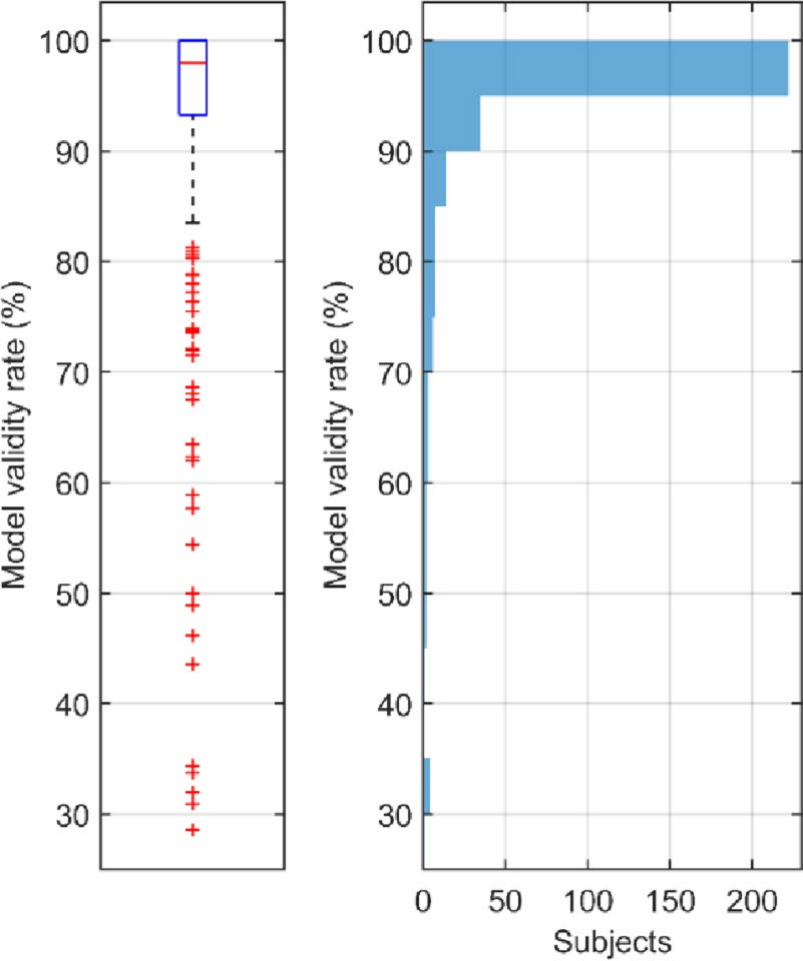
Boxplot and histogram of the model validity rate.

All sequences from the HSS database were processed according to the outlined methodology. First, the validity of the proposed models for both GD and PAV have been evaluated by computing the percentage of valid model fits per subject, where a model fit is considered valid if the four coefficients of determination (two for the fittings to Eqs. (2) and (3) for both GD and PAV) were greater than 0.8. A reported median validity of 98% and an interquartile range of 8% suggests that the model performs well for most participants. Figure 2 shows the boxplot and histogram with the results of the model validity rate.

The presence of outliers in the distribution raises questions about potential inconsistencies in the data or the model’s application. One possible explanation for these outliers is natural variability among participants, meaning that certain individuals inherently exhibit patterns that the model fails to capture adequately. However, another plausible reason is the presence of measurement errors or issues in the algorithm that processes the data. If raw data contain noise, artifacts, or systematic errors, this could lead to miscalculations in the extracted model parameters, making some fits appear invalid when, in reality, the issue lies in the data quality. Similarly, inconsistencies in the algorithm responsible for extracting the parameters from the measurements—such as misalignment, incorrect preprocessing, or computational biases—can artificially introduce extreme values in some participants, falsely inflating the number of outliers.

To establish baseline parameters for GD and pupil dynamics during saccadic tasks in a healthy population that allow investigating hemispheric differences and assessing interrelations, a statistical assessment of the available data was conducted. Participants were excluded from the analysis if they had fewer than 40 correctly recorded target changes or a model validity rate below 70%. Subsequently, 20 rightward (RW) changes, target shifting from −15 to + 15 dva, and 20 leftward (LW) changes were randomly selected. As a result, the number of balanced participants was reduced to 242, yielding a total of 9680 eye saccadic and PSO subsequences.

The parameters derived from the fits to Eqs. (2) and (3) exhibited significant deviations from normality (Shapiro–Wilk test, *p* < 0.01). Consequently, Wilcoxon signed-rank tests were employed to evaluate differences between RW and LW shifts. The null hypothesis was rejected for all parameters (*p* < 0.01), with high W statistics indicating robust asymmetries between the two shift directions.

As a result of testing different probability density functions, the TLS distribution emerged as the most frequent optimal fit, ranking first for 55.6% of the metrics. Even in the remaining cases where other non-normal distributions performed better, the ∆AIC compared to a standard Gaussian model was consistently over 1000. This represents an overwhelming rejection of the normality assumption, given that a ∆AIC greater than 10 is usually considered decisive^48^. Consequently, we used Medians and interquartile ranges (IQR) for descriptive statistics. For our comparative analysis, we performed Wilcoxon signed-rank tests using subject-level summary statistics; for each of the 242 participants, the median value for leftward movements was paired against the median for rightward movements. This ensured the tests were robust to the observed outliers while strictly honoring the paired nature of the data. Computed medians and IQR for LW and RW shifts for the GD and PAV are respectively shown in Tables 1 and 2.

**Table 1:** Saccade parameters. Median values, interquartile range (IQR), and p-values (p) for saccade parameters. Results correspond to the parameters of Eq. (2) for both GD and PAV across LW and RW target shifts. Data are presented as Median [25th, 75th percentiles]. p-values were calculated using the Wilcoxon signed-rank test to assess directional asymmetries, with pairing performed at the subject level.

		Saccade							
		*b* (s^−1^)	*p*	*c* (ms)	*p*	*a* (dva)	*p*	*d* (dva)	*p*
GD	LW	77 [70, 84]	< 0.001	13 [10, 15]	< 0.001	30.5 [30.0, 31.1]	0.073	−14.3 [−14.8, −13.8]	< 0.001
	RW	74 [67, 84]		10 [5, 13]		30.4 [29.8, 31.0]		−16.0 [−16.4, −15.7]	

		Saccade							
		*b* (s^−1^)	*p*	*c* (ms)	*p*	*a* (a.u.)	*p*	*d* (a.u.)	*p*
PAV	LW	197 [155, 284]	< 0.001	3 [−1, 6]	< 0.001	−3.5 [−3.7, −3.3]	0.023	0.59 [0.37, 0.83]	0.004
	RW	170 [120, 283]		2 [−11, 5]		−3.5 [−3.8, −3.1]		0.46 [0.25, 0.71]	

**Table 2:** Medians and IQR of the parameters of the fittings to Eq. (3) of the post saccadic GD and the PAV when the LW and RW target shifts.

		PSO									
		*A* (dva)	p	*δ* (ms)	p	*T* (ms)	p	*t*_0_ (ms)	p	*h* (dva)	p
GD	LW	0.25 [0.14, 0.36]	< 0.001	9 [7, 11]	< 0.001	34 [29, 43]	0.005	65 [59, 70]	0.080	14.0 [13.4, 14.6]	0.522
	RW	0.11 [0.03, 0.22]		7 [6, 9]		36 [31, 48]		64 [57, 71]		13.9 [13.4, 14.5]	

		PSO									
		*A* (a.u.)	p	*δ* (ms)	p	*T* (ms)	p	*t*_0_ (ms)	p	*h* (a.u.)	p
PAV	LW	0.57 [0.36, 0.86]	0.009	40 [31, 52]	< 0.001	96 [86, 104]	< 0.001	76 [71, 83]	< 0.001	0.12 [−0.07, 0.32]	< 0.001
	RW	0.58 [0.31, 0.80]		31 [25, 39]		89 [81, 98]		73 [68, 77]		0.32 [0.15, 0.54]	

Time parameters are the most meaningful for comparing gaze and pupil dynamics. The parameter $\:c$ is the instant when the sigmoid function reaches the half-height, $\:a$ determines the height of the curve, $\:b$ controls the steepness of the curve and $\:d$ is the baseline. Regarding the PSO, $\:A$ determines the maximum height of the oscillation at $\:t={t}_{0}$, the parameter $\:{t}_{0}\:$sets the starting point of the oscillation, $\:T$ is the period of the oscillation, $\:\delta\:$ is the damping term and $\:h$ is the baseline. The missing parameter $\:g$ represents a linear increase. This parameter does not provide relevant information but serves as a mathematical construct necessary for achieving a better fit to the function.

Additional time parameters can be defined to provide a more detailed characterization the movements dynamics. From $\:b$, it can be defined the parameter $\:{t}_{10-90}=4.5/b$, which determines the time it takes to transition from 10% to 90% the amplitude of the shift. The initiation and termination points of the saccade can be defined for both GD and PVA as $\:{t}_{si}=c-\frac{{t}_{10-90}}{2}$ and $\:{t}_{sf}=c+\frac{{t}_{10-90}}{2}$. The endpoint of the PSO is determined as the moment when 10% of the initial amplitude is reached. This time is then given by the equation $\:{t}_{pf}={t}_{0}-\delta\:\mathrm{ln} \left(0.1\right)$. Finally, by considering saccadic movement and PSO together, the movement duration for pupil size variation and GD was estimated as $\:\varDelta\:t={t}_{pf}-{t}_{si}$. Table 3 shows the computed median and IQR. Again, none of the dataset conformed to a normal distribution, so the Wilcoxon signed-rank test was used to compare paired sets of samples (GD vs. PAV and LW vs. RW). The analysis revealed that most parameters exhibited significant directional asymmetries. Specifically, the total duration of gaze displacement ($\:\varDelta\:t$) showed a highly significant difference between directions (*p* < 0.001), whereas the total duration for the pupillary response remained relatively balanced (*p* = 0.285).

**Table 3:** Temporal characteristics of saccadic and PSO for GD and PAV.

		*t*_*si*_ (ms)	p	*t*_*sf*_ (ms)	p	*t*_*pf*_ (ms)	p	∆*t* (ms)	p
GD	LW	−17 [−21, −14]	< 0.001	41 [35, 46]	0.006	87 [78, 95]	< 0.001	104 [97, 112]	0.285
	RW	−20 [−27, −17]		39 [33, 44]		82 [76, 90]		105 [97, 114]	
PAV	LW	−9 [−17, −3]	< 0.001	13 [10, 15]	0.096	173 [153, 205]	< 0.001	186 [164, 240]	< 0.001
	RW	−14 [−31, −4]		12 [9, 16]		149 [131, 179]		167 [146, 202]	

In contrast to the temporal initiation shifts, several parameters demonstrated directional symmetry. No significant differences were observed between leftward and rightward movements for the amplitudes (*a*) of both GD and PAV, or the final pupillary stabilization time ($\:{t}_{sf}$) (*p* > 0.01). Specifically regarding gaze dynamics, the baseline offsets ($\:{t}_{0}$, *h*) also remained invariant to direction (*p* > 0.01), indicating a consistent physical starting point for the eyes regardless of the intended shift direction.

**Fig. 3:**
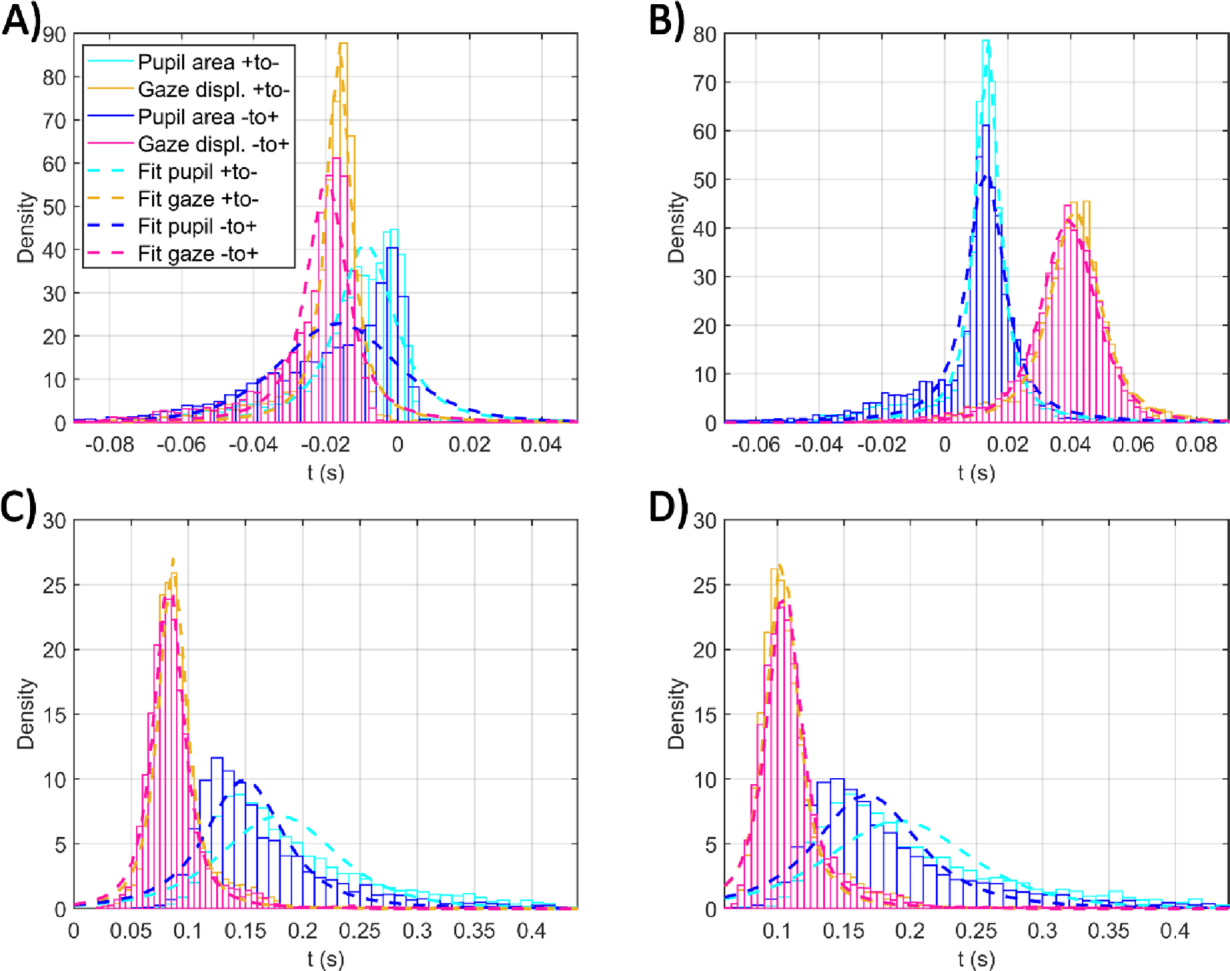
Population distributions and TLS model fits. Density plots for the temporal parameters $\:{t}_{si}$, $\:{t}_{sf}$, $\:{t}_{pf}$ and $\:\varDelta\:t$ are shown in **A**–**D**, respectively. The histograms represent the empirical density of the observed data, while the solid curves represent the fitted TLS distributions. The TLS model was specifically selected for its ability to handle heavy-tailed data and outliers; which results in the smooth curves seen over the empirical histograms.

Densities of the additional time parameter were fitted to TLS distributions. The appropriateness of the TLS model was confirmed by the low estimated degrees of freedom ($\:\nu\:$), which typically ranged between 1.5 and 4.0. These low $\:\nu\:$ values indicate a heavy-tailed distribution where outliers are significantly more probable than in a standard Gaussian framework. Figure 3A–D respectively represent these distributions of the parameters $\:{t}_{si}$, $\:{t}_{sf}$, $\:{t}_{pf}$ and $\:\varDelta\:t$ .

## Discussion

In this study, sequences of eye movements when performing saccadic tasks from an extensive existing database from the literature have been analysed. A sigmoidal Boltzmann function and a damped harmonic oscillator were proposed to respectively model saccades and post saccadic oscillations and sequences from 242 participants were processed. Rightward and leftward GD and PAV have been modelled, extracting temporal characterizing parameters that can serve to establish reference values for those conditions.

This work revealed that PFE masks genuine changes in pupil area. Saccades and PSO can also be identified in the registered PAV, and they can be modelled similarly to GD. To the best of our knowledge, this is the first time it has been reported in the literature. Previous studies have demonstrated pupil responses during saccades, although they primarily focus on assessing later time periods^49,50^. Although the latencies associated with the target change were not directly measured, the results presented in Table 3 suggest that pupil dilation begins slightly before the onset of saccadic eye movement and concludes considerably earlier than the completion of the GD. Finally, bearing in mind that the PSO for pupil area initiated at $\:t=0$ s, gaze position PSO stabilizes faster than pupil area ones. Moreover, considering saccadic movement and PSO the overall duration of the phenomenon was longer for the pupil.

The analysis revealed distinct directional asymmetries. The total duration ($\:\varDelta\:t$) of GD was significantly longer for LW movements than for RW ones (186 ms vs. 167 ms, *p* < 0.001). In contrast, while the PAV followed a similar numerical trend, the difference in its total duration did not reach statistical significance (*p* = 0.285). These findings are provides strong empirical support for previous reports^17,18^ regarding slower adaptation in LW saccades, a pattern that remains robust when analyzed at the subject level. That pattern is observed in all time points presented in Table 2, not only the total interval. Consequently, RW movements started and ended earlier than LW ones, a temporal shift that was consistently detectable within the proposed modeling framework.

Interestingly, while temporal initiation and the peak response showed clear directional biases, several parameters remained invariant to direction (p > 0.01). Specifically, the amplitudes (*a*) for both GD and PAV and the final pupil stabilization time ($\:{t}_{sf}$) showed no significant differences between LW and RW movements. This symmetry extended to the gaze baseline offsets ($\:{t}_{0}$, $\:h$), suggesting that the physical ‘launch point’ of the eye is consistent. This suggests a functional decoupling: while ‘pre-saccadic’ preparation and timing are lateralized, potentially reflecting cortical dominance or habitual reading patterns, the total mechanical energy (amplitude) and the ultimate settling of the autonomic response appear to be governed by symmetrical homeostatic constraints. The lack of a significant difference in $\:\varDelta\:t$ for GD further implies that the internal clock governing the total duration of the gaze-pupil coordination window is conserved, ensuring a consistent functional window regardless of the direction of the shift.

With respect to PSO, the period for GD oscillations was shorter than for pupil size variation (see Table 1). Previous studies on PSO frequencies in populations of fewer than 10 participants have reported ranges between 15 and 20 Hz^24^ and 30–40 Hz^51^. These findings align with the PSO frequencies observed for GD in this study of 242 participants, which were approximately 29 Hz. The variation in pupil area oscillated during the PSO at a frequency of 11 Hz.

The high-precision characterization of the *b* and *T* parameters provides new insight into the biomechanical ‘ringing’ of the eye. In oculomotor literature, PSO and glissades are often attributed to the viscoelastic properties of the extraocular muscles and the dynamic mismatch between the neural pulse and step commands^19,52^. By capturing these dynamics within a unified framework, our results suggest that this mechanical ‘overshoot’ is a consistent signature of the oculomotor plant’s damping characteristics. Recent evidence suggests that these oscillations are not merely motor noise but can significantly influence the time required for subsequent visual processing^21,55^. The significant directional asymmetry observed (p < 0.001) suggests that the neural ‘pulse’ and the mechanical resistance of the orbital tissues may be tuned differently depending on the direction of displacement.

It is important to distinguish between PSOs as a pure biomechanical rotation of the eyeball and as they are expressed in pupil-based eye-tracking signals. In video-based systems, the recorded oscillation often reflects the inertial ‘wobble’ of the lens and pupil relative to the cornea. While our analysis focuses on large 30° saccades to ensure a high signal-to-noise ratio, evidence suggests that PSOs do not necessarily scale linearly with amplitude. In many cases, shorter saccades involve a more abrupt deceleration or ‘harder braking’ compared to the relatively gentler stabilization seen in larger 30° movements. This implies that the oscillations we observed may actually be more pronounced during the smaller, more frequent saccades typical of naturalistic viewing. Consequently, the 30° paradigm used here likely represents a conservative baseline, suggesting that the functional relevance of these oscillations for visual processing is likely even more significant in everyday oculomotor behavior.

Furthermore, the ‘temporal decoupling’ observed—where the pupil recovery window ($\:\varDelta\:t$) shows significant asymmetry while the macroscopic gaze duration does not—extends current understanding of peri-saccadic pupil modulation. While pupillary changes are often viewed as a general proxy for arousal^53^, our results suggest a more localized relationship where the pupil reflects the ‘computational cost’ or neural effort required by brainstem circuits to stabilize the gaze after a large-amplitude shift. This aligns with evidence that the superior colliculus and the locus coeruleus coordinate both saccadic execution and pupillary diameter^49,54^. By bridging these dynamics, the current framework positions joint gaze-pupil signatures as sensitive biomarkers for detecting lateralized brainstem or autonomic dysfunctions that might be overlooked by traditional metrics.

The robustness of these findings is supported by our systematic model selection. Across the dataset, parameters were best described by a TLS distribution, as indicated by the lowest AIC values. The deviation from a normal distribution—evidenced by $\:\varDelta\:$AIC values consistently exceeding 1000—highlights the presence of heavy tails and outliers inherent in large-scale oculomotor data. While the TLS distribution provided the best fit overall, the low estimated degrees of freedom ($\:\nu\:$) in certain parameters indicate extreme dispersion where variance may not be well-defined. This complex population structure justifies our move away from Gaussian assumptions toward robust non-parametric techniques, such as the Wilcoxon signed-rank tests used for our comparisons.

Despite the high-precision fit of the unified GD-PAV model, its current application has specific boundaries. These results were obtained under head-fixed conditions during enforced 30° horizontal saccades; while this was necessary to isolate the fine-scale dynamics of the oculomotor plant, the behavior may differ in naturalistic viewing where head-gaze coordination and varying amplitudes introduce additional complexity. In everyday behavior, saccades of this magnitude are relatively uncommon and are typically compensated by synergistic head movements. By constraining the head, our paradigm necessitates a higher degree of orbital displacement and ‘harder’ braking by the extraocular muscles, which likely amplifies both the magnitude of the PSO and the associated pupillary response. Consequently, while the results provide a robust baseline for the oculomotor plant’s limits, they should be interpreted as a ‘maximum-load’ scenario rather than a direct representation of unconstrained exploratory gaze shifts. While PSO characteristics are known to scale with saccade amplitude, the use of a fixed 30° benchmark was intentional to characterize directional asymmetries (LW vs. RW) across a large population without the confounding influence of amplitude-dependent variance. This standardized approach ensured that the observed temporal shifts and model parameters were attributable to the directional control of the oculomotor system rather than fluctuations in movement scale. Future work should evaluate how these results are influenced by different directions and amplitudes to see if the observed asymmetries scale linearly across the broader saccadic ‘main sequence.’ Additionally, there is clear clinical interest in testing this framework in populations with neurological disorders^25,39–45^. By establishing this high-precision baseline, we provide a foundation for future research to explore the neural effort and somatic-autonomic coordination of human vision in increasingly complex, unconstrained scenarios.

To further contextualize the performance of the proposed unified framework, a brief comparison with related approaches is warranted. The most directly comparable precedent for modeling PSO in gaze data is the damped harmonic oscillator model employed by Guadron et al.^25^, which was applied to a clinical population of 18 participants (6 AMD, 5 RP, 7 controls) using the same parametric form as Eq. (3). In contrast, the present study extends this approach to a healthy normative population of 242 participants, yielding substantially more robust distributional estimates. Whereas Guadron et al. reported PSO frequencies of approximately 10–15 Hz in healthy controls, our GD-based estimates converge at approximately 29 Hz, a discrepancy consistent with the known dependence of PSO frequency on saccade amplitude and the different tracker configurations used. Tabernero and Artal^24^, using a dual Purkinje image tracker, reported lens oscillation frequencies of approximately 20 Hz for 4° saccades, a value expected to be lower than the 30° saccades analyzed here given the nonlinear scaling of PSO with amplitude. Regarding saccade modeling, prior work employing sigmoidal functions has typically been applied to individual trials or small samples without characterizing population-level parameter distributions. The key methodological advance of the present framework is the simultaneous modeling of both GD and PAV under a unified parametric scheme applied to a large normative dataset, combined with robust non-Gaussian (t location-scale) population modeling. This distinguishes the approach from single-signal, small-sample predecessors and provides the reference values necessary for future clinical benchmarking against pathological populations.

## Data Availability

This study uses data from the Horizontal Saccade Task (HSS), collected from 309 participants in the GazeBase dataset (licensed under CC BY 4.0), as described by Griffith, H., Lohr, D., Abdulin, E. & Komogortsev, O. “GazeBase, a large-scale, multi-stimulus, longitudinal eye movement dataset,” Sci Data 8, 184 (2021). The extracted eye movement sequences and derived parameters used in this work are publicly available at the Institutional Repository of the University of Alicante, http://hdl.handle.net/10045/155507.

## Code availability

The custom MATLAB code used to implement the PFE correction procedure, the curve-fitting routines for the Boltzmann sigmoid and damped harmonic oscillator models (Eqs. 2 and 3), the quality filtering and trial-balancing pipeline, the population distribution fitting, and the subject-level statistical comparisons is publicly available at the Institutional Repository of the University of Alicante: http://hdl.handle.net/10045/164099. The MNH saccade detection algorithm applied upstream was used as originally described by Friedman et al.^30^ and Nyström and Holmqvist^31^; no modifications were made to that algorithm. All analyses were performed using MATLAB (R2020b or later) with the Curve Fitting Toolbox, the Statistics and Machine Learning Toolbox, and the Signal Processing Toolbox.
